# Serum anti-p53 antibody as a tumour marker for colorectal cancer screening

**DOI:** 10.3332/ecancer.2015.560

**Published:** 2015-07-29

**Authors:** Masaya Iwamuro, Yoshinari Kawai, Tomoko Matsumoto, Masashi Uda, Hiroyuki Okada

**Affiliations:** 1 Department of Gastroenterology and Hepatology, Okayama University Graduate School of Medicine, Dentistry, and Pharmaceutical Sciences, Okayama 700-8558, Japan; 2 Department of Gastroenterology, Onomichi Municipal Hospital, 722-8503, Onomichi, Japan; 3 Department of Surgery, Onomichi Municipal Hospital, 722-8503, Onomichi, Japan; 4 Department of Endoscopy, Okayama University Hospital, Okayama 700-8558, Japan

**Keywords:** anti-p53 antibody, colon cancers, colorectal carcinoma, cancer screening tests, early diagnosis of cancer

## Abstract

A 60-year-old Japanese man presented to our hospital for further investigation of an elevated serum anti-p53 antibody level. He was diagnosed with colon cancer and the tumour was surgically resected. Histological diagnosis of advanced colon cancer without lymph node involvement or distant metastasis was made. It was noteworthy that both serum carcinoembryonic antigen (CEA) and a fecal occult blood test that were performed preoperatively were non-diagnostic. This case highlights the potential usefulness of serum anti-p53 antibody tests for detection of colorectal cancers. Moreover, sequential changes in the anti-p53 antibody levels after curative resection were observed.

## Introduction

p53, a well-known tumour suppressor protein, is frequently mutated in various kinds of malignant diseases during tumourigenesis. For example, mutation of the p53 gene occurs as a critical event in the progression from adenoma to adenocarcinoma in the colorectum [1–3]. Accumulation of mutated p53 protein can be observed in the malignant cells, because the half-life of the mutated p53 protein is longer than that of the wild-type p53. Such accumulation subsequently causes an immune response against the mutated p53 protein because of a self-immunisation process linked to the strong immunogenicity of the mutated p53 protein [4]. Previous reports revealed that 13–32% of patients with colorectal cancers are positive for anti-p53 antibodies [5–7].

Since mutation of p53 is involved in the early stages of cancer development, anti-p53 antibody has been investigated as one of the more promising candidate markers for early detection of colorectal cancers. In this report, we present a case of limited-stage colon cancer. Though CEA was not elevated and the patient had no symptoms, a positive anti-p53 antibody test prompted him to undergo further investigations. The potential utility of anti-p53 antibody in the management of colorectal cancer patients is discussed. Another important finding from the present patient is that it took 21 months to normalise anti-p53 antibody levels after resection of the colon cancer. Sequential changes of anti-p53 antibody levels after curative treatment are described.

## Case report

A 60-year-old Japanese man presented to Onomichi Municipal Hospital for further investigation of an elevated serum anti-p53 antibody level. The patient had been taking no medications, and he had no history of gastrointestinal diseases. He had been undergoing annual fecal occult blood test and also blood tests which included tests for anti-p53 antibodies and CEA, as routine medical check-ups. Blood tests for anti-p53 antibodies and CEA are optional during an annual health checkup at the patient’s workplace, and these tests were included upon his request. Although his serum anti-p53 antibody level one year previously was 0.0 U/mL, the level had risen to 11.5 U/mL (normal range: 0.0–1.3 U/mL). CEA was within the normal range. A fecal occult blood test performed six months earlier was negative.

**Figure 1.**
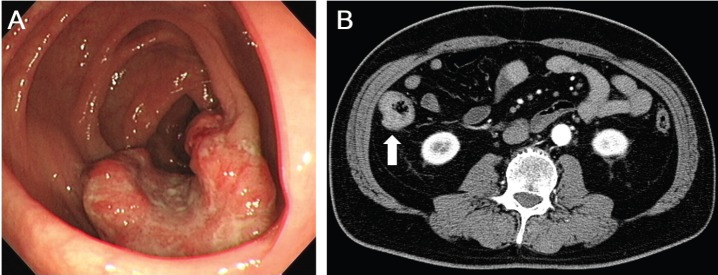
Images of the ascending colon with a cancerous lesion. (A) Colonoscopy examinations show an ulcerated tumour in the ascending colon. (B) CT scanning with contrast media reveals a mucosal thickness in the ascending colon without lymph node involvement or metastatic tumours.

The patient had no symptoms. Physical examination revealed no abnormalities, and there was no evidence of an abdominal mass or peripheral lymphadenopathy. Laboratory tests revealed hyperglycaemia and hyperlipidaemia. Colonoscopy demonstrated an ulcerated tumour in the ascending colon (Figure 1A). Moderately differentiated adenocarcinoma was detected in the biopsy specimen taken from the tumour. Computed tomography (CT) scanning of the neck, chest, abdomen, and pelvis revealed a mucosal thickness in the ascending colon, but there were no lymph node swellings or metastatic lesions (Figure 1B). Consequently, the diagnosis of colon cancer, localised in the colon, was made.

**Figure 2.**
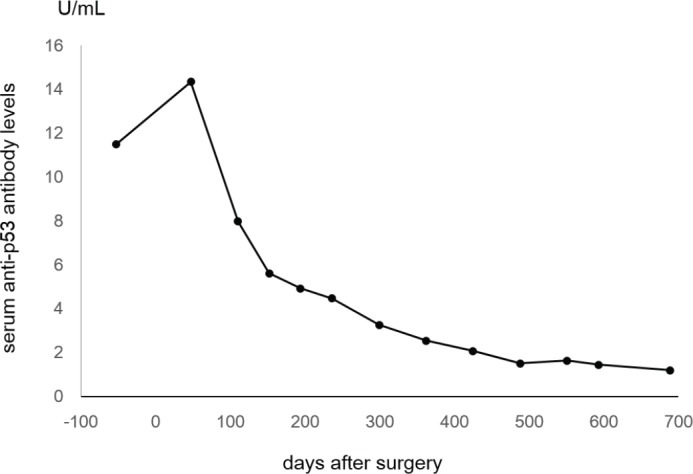
Line chart showing changes of serum anti-p53 antibody levels. The levels had gradually been decreasing and finally normalised 689 days after the surgical resection.

The tumour was surgically resected along with peripheral lymph node excision. The postoperative diagnosis was stage II colon cancer. Pathological assessment revealed moderately differentiated tubular adenocarcinoma with subserosal invasion. Metastasis was not detected in the dissected lymph nodes. The patient recovered uneventfully after the surgery. Forty-seven days after the surgery, the serum level of anti-p53 antibody was 14.34 U/mL. The levels had gradually been decreasing, and it finally normalised 21 months after the curative resection (Figure 2).

## Discussion

The p53 gene is a well-known tumour suppressor gene, and mutation of this gene is a critical event in the carcinogenesis of various types of tumours [8–10]. Although the mechanisms of autoantibody production against p53 have not fully revealed, it is thought to be linked to p53 gene missense mutations that lead to the accumulation of p53 protein in the tumour [4, 5]. The potential clinical utility of serum anti-p53 antibodies was first described in 1982 by Crawford *et al* [5, 11]. They reported that anti-p53 antibodies were not detected among 164 healthy subjects, whereas 14/155 patients (9.0%) with breast cancer were positive. Soussi *et al* reviewed previous studies and reported that serum anti-p53 antibodies were detected in 16.8% of patients with solid tumours (1600/9489 cases) and only 1.5% of healthy controls (35/2404 individuals) [4]. Although the sensitivity of serum anti-p53 antibody is only 20–40% in patients with solid tumours with p53 mutations, the specificity is approximately 95% [12]. Therefore, it has been proposed as a biomarker for early detection of diseases in cancer screening.

For colorectal cancer screening, Pedersen *et al* retrospectively examined the development of p53 autoantibodies in a large-scale study in the general population [12]. They investigated stored serum samples of 50,640 women who had enrolled in an ovarian cancer screening study where annual blood samples were collected [12]. Of these, 97 patients were diagnosed with colorectal cancer after enrolling in the study. A combination of four p53 peptides (p53–9, –10, –25, and –78) identified 26% of the colorectal cancer patients in this cohort. Moreover, anti-53 antibodies could be detected in the serum samples collected 1.4 years before the clinical diagnosis. Pedersen *et al*’s report is the first cohort study revealing the usefulness of anti-p53 antibodies for early detection of colorectal cancers.

In the present patient, elevated serum levels of anti-p53 antibodies were the only sign of colon cancer because the patient had no symptoms, CEA was within the normal range, and the fecal occult blood test was negative. This report reinforces the value of anti-p53 antibody testing for colorectal cancer screening.

A relationship between curability by surgical resection and postoperative disappearance of anti-p53 antibodies has also been reported [13–17]. Takeda *et al* evaluated serum anti-p53 antibody levels before and after resection of colorectal cancer in 40 patients [18]. They found a significant correlation between curability and postoperative disappearance of the autoantibodies. Development of anti-p53 antibodies in superficial colorectal cancer patients and disappearance of the antibodies after endoscopic resection have been reported by the same group [1]. Total disappearance of anti-p53 antibodies in association with a favourable response to therapy have been reported in other solid tumours as well [19, 20].

In the present patient, negative conversion of anti-p53 antibodies after curative surgical resection was observed. It is noteworthy that the levels of anti-p53 antibodies gradually decreased after resection, and it took 21 months to become normalised (Figure 2). Gradual decreases of anti-p53 antibody levels after the completion of treatment have been described previously [14]. Therefore, physicians should take into consideration that negative conversion of anti-p53 antibodies requires a longer time, even after curative treatment, compared with other conventional tumour markers such as CEA.

## Conclusions

We treated a patient with colon cancer. A positive serum anti-p53 antibody result prompted him to undergo further investigation that led to the diagnosis. Although the sensitivity and the specificity need to be investigated in a prospective study, serum anti-p53 antibody may be a useful tumour marker for colorectal cancer screening.

## List of abbreviations

CEA: carcinoembryonic antigen
CT: Computed tomography

## Conflicts of interest

The authors state that they have no conflict of interest.
